# Evaluation of risk factors for insulin resistance: a cross sectional study among employees at a private university in Lebanon

**DOI:** 10.1186/s12902-020-00558-9

**Published:** 2020-06-10

**Authors:** Myriam Fahed, Maya G. Abou Jaoudeh, Samar Merhi, Jocelyne Matar Bou Mosleh, Rachelle Ghadieh, Sibelle Al Hayek, Jessy E. El Hayek Fares

**Affiliations:** 1 Department of Nursing and Health Sciences, Notre Dame University- Louaize (NDU), Zouk Mosbeh, Lebanon; 2 Department of Endocrinology, Diabetes, Metabolism and Eating Disorders, University Hospital of Saint-Etienne, Saint-Etienne Cedex, France; 3 Department of Dietetics and Nutrition, The University of Kansas Medical Center, 3901 Rainbow Blvd, Kansas City, KS 66160 USA

**Keywords:** Dairy products, Insulin resistance, Homeostatic model assessment for insulin resistance, Cross-sectional, Human subjects

## Abstract

**Background:**

Worldwide, the prevalence of insulin resistance ranges from 15.5 to 46.5%, among adults. Lebanon reported one of the highest rates reaching 44.6%. The literature suggests an association between dairy product consumption and insulin resistance, however results are inconclusive. To our knowledge, no study examined this association in the Middle Eastern Region. The aim of the study was to investigate the prevalence of insulin resistance among a sample of Lebanese adults, to identify its risk factors depending on gender, and to evaluate the association between insulin resistance and dairy products consumption.

**Methods:**

A cross-sectional study was conducted among employees at Notre Dame University - Louaize. Four questionnaires were administered including a background and International Physical Activity Questionnaire short form questionnaires, food frequency questionnaire and a 24 h recall. Bioelectric Impedance Analysis (BIA) was used to measure percent body fat (PBF). Homeostatic Model Assessment for Insulin Resistance (HOMA-IR) was used to quantify insulin resistance. A person with HOMA-IR ≥ 2.5 was considered as insulin resistant. Statistical analyses were performed using the Statistical Package for Social Sciences version 23 for Windows. *P* < 0.05 was considered statistically significant.

**Results:**

Out of 286 study participants, 38.0% were insulin resistant. Average dairy product intake in the total sample was 2.2 ± 1.0 servings per day. Among males, the odds of having insulin resistance were 3.9 times higher (95%CI 1.4–11.0; *p* = 0.009) for those having a risky waist circumference compared to those having a healthy waist circumference. Among females, being married (OR: 0.2, 95%CI 0.1–0.5; *p* = 0.002), PBF (OR: 1.2, 95%CI 1.0–1.3; *p* = 0.008) and hypertriglyceridemia (OR: 8.7, 95%CI: 2.1–35.9; *p* = 0.003) were associated with HOMA-IR, after controlling for confounders. Dairy intake was not associated with HOMA-IR neither among males (*p* = 0.777), nor among females (*p* = 0.968), after controlling for confounders.

**Conclusion:**

Dairy consumption was not associated with increased insulin resistance. More research focusing on the relationship between dairy intake and insulin resistance is needed, especially in the Arab and Middle-Eastern region. Future studies should examine the effect of different types of dairy products and the effect of different nutrients in dairy products on insulin resistance.

## Background

According to the International Diabetes Federation, the number of people with diabetes in the world is expected to increase from 463 million in 2019 to 700 million in 2045, with 83.9% of cases occurring in low and middle income countries [1]. In Lebanon, World Health Organization (WHO) statistics showed that diabetes is standing as the fourth leading cause of death (4%) with a prevalence rate of 12.6% [2], higher than that reported by the United States (US) (9.4%) [3].

Type 2 diabetes, the most common form of diabetes, is mainly caused by insulin resistance [4]. Insulin resistance can be defined as a condition in which the pancreas is required to secrete more insulin than normal in order to achieve normal blood glucose levels due to reduced sensitivity or responsiveness of tissues to insulin biologic activity [5]. The prevalence of insulin resistance varies across countries. Studies showed that it is estimated to be the lowest among European adults with a prevalence of 15.5% [6], while higher prevalence rates were reported in other countries reaching 23.3, 39.1 and 46.5% in Thailand, Texas-US and Venezuela [7–9], respectively. Lebanon reported one of the highest prevalence rates compared to other countries reaching 44.6% among a national sample of 308 adults with an average age of 41.0 ± 15.5 years [10].

Several factors could increase the likelihood to develop insulin resistance. For instance, age increases the risk of having insulin resistance due to the high proportion of visceral fat, oxidative stress and mitochondrial dysfunction [11, 12]. Abdominal adiposity and increased body fat are other risk factors for insulin resistance [13, 14] and this is due to the high amount of free fatty acids and pro-inflammatory cytokines released from visceral fat tissue into the portal vein of obese subjects, causing the development of hepatic insulin resistance and type 2 diabetes [15]. Other risk factors include gender [16–18], physical inactivity [19, 20].

Further, diet has been shown to be effective in improving insulin resistance [21] and reducing the incidence of type 2 diabetes [22]. A study conducted among subjects with impaired glucose tolerance showed that diet was able to reduce the incidence of type 2 diabetes by 33% (*p* < 0.003), after a follow up period of 6 years [22]. In a study conducted among overweight and obese middle aged women, diet alone was able to reduce insulin resistance by 24% [21]. Accordingly, more research is focusing on the role of specific food groups in improving insulin resistance.

The consumption of dairy products makes an important contribution to the human diet. They were found to provide more calcium, protein, magnesium, potassium, zinc, and phosphorus per calorie than any other type of food [23]. In Lebanon, as well as in other countries in the Levant region such as Jordan and Syria, dairy products constitute an important part of the traditional food heritage, typically in the fermented form like yogurt, labneh and white cheese [24]. However, not only in Lebanon, but among all Middle Eastern countries, globalization has led to a decreased consumption of dairy products over time [25, 26] and a shift to a Western diet including high consumption of fast foods as a result of rapid economic and social changes. These dietary changes were found to be major contributors to central obesity and insulin resistance resulting in an increase in the epidemic of type 2 diabetes [27].

Typical predictors of insulin resistance such as age, gender, physical inactivity, abdominal adiposity and body fat mass have been studied thoroughly in the literature, yet other factors such as dairy products were understudied. To date, few epidemiological studies examined the association between dairy product consumption and insulin resistance; and results are still controversial and require further assessment [28–34]. To our knowledge, no study has examined this association in the Arab and Middle Eastern Region, particularly in Lebanon. It is likely that this association could be different by ethnicity due to different genetic predisposition for diabetes [35]. All of these factors highlight the importance of examining this association in Lebanon. Accordingly, the aim of the study was to investigate the prevalence of insulin resistance among a sample of Lebanese adults, to identify its risk factors depending on gender, and to evaluate the association between insulin resistance and dairy products consumption.

## Methods

### Study design and recruitment methods

A cross-sectional study was carried out on Notre Dame University (NDU) employees in the Zouk Mosbeh, North, and Shouf campus between October 2016 and March 2017. The detailed study methodology was previously published [36]. Taking into account the prevalence of insulin resistance of 44.6% in the Lebanese population [10], a confidence interval of 95% and a margin of error (d) of 5.5%, the calculated sample size was estimated to be 316 participants.

Among 600 contacted employees in the three NDU campuses, 360 accepted to participate. Exclusion criteria included pregnancy and lactation, cardiovascular disease (stroke, heart failure and heart attack), diabetes, failure to complete the questionnaires, and presence of a pacemaker or metal pieces in the participant’s body. Those who were found to be eligible (*n* = 286) were contacted by the study investigators to arrange for a 30-min face-to-face interview. All subjects signed informed consent before participating in the study. The study protocol was approved by the Institutional Review Board of Notre Dame University and performed in accordance with the Helsinki Declaration.

### Data collection procedures

#### Questionnaires

During the 30-min face-to-face interview, trained nutritionists filled out four questionnaires: background questionnaire, International Physical Activity Questionnaire, food frequency questionnaire, and a 24-h Multiple-Pass Method recall. All questionnaires were pre-tested using a sample of thirty NDU employees in the three campuses. Revisions and corrections were performed before initializing the study. Details about the background questionnaire (available as a supplementary file [Additional file 1]) and International Physical Activity Questionnaire [37] were previously described [36].

#### Food frequency questionnaire (FFQ)

Dairy products intake was assessed using a FFQ that was previously developed by study investigators [38] and adapted to the Lebanese population, including different types of dairy products (milk, yogurt, cheese, ice cream, and labneh). For each food item, participants were asked to mark their frequency of intake of a designated serving/portion size per day/week/months or rarely/never during the past year.

#### 24-h multiple-pass method recall

A 24-h Multiple-Pass Method recall was used to estimate energy and nutrient intake. It consisted of a five-step approach conducted in person by a trained nutritionist. Step 1 started with a Quick List where respondents list all foods consumed in a 24-h period. Step 2 included a series of questions that probe for foods that were commonly forgotten during Step 1 including beverages, alcoholic beverages, sweets, savory snacks, fruits, vegetables or cheese, breads or rolls, and one for any additional food not previously mentioned. Step 3 collected the time each food was eaten and the eating occasion. Step 4 was the Detail Cycle where descriptions were obtained for each food reported, along with quantities consumed and where the food was consumed. Step 5 was a final review question, the Final Probe, which provided the respondent a last opportunity to recall any foods that had not been previously reported in the interview [39].

#### Energy and nutrient intake

The Nutritionist Pro diet analysis software, version 31.0 (Axxya Systems, Woodinville, WA, US), was used to create estimates of energy and different nutrients’ intake.

#### Anthropometric measurements

Anthropometric measurements including height, waist circumference, body weight were taken and body composition was assessed using BIA machine InBody 720 (Biospace, Seoul, Korea). Details about anthropometric measurements was previously published [36].

#### Biochemical measurements

Upon the visit to the nutrition laboratory, a nurse collected a fasting sample of blood. Samples collected at the Shouf and North campuses were transported to the Zouk Mosbeh campus on ice. Samples were stored at -20 °C in the freezer for a maximum of 6 weeks before analysis. Biochemical assessment included serum triglycerides (TG), total cholesterol, high density lipoproteins (HDL) cholesterol, low density lipoproteins (LDL) cholesterol, c-reactive protein (CRP), and fasting blood glucose which were measured using a dry chemistry analyzer Vitros 250 (Ortho Clinical Diagnostic, Raritan, New Jersey, US) available at the Biology laboratory at the Zouk Mosbeh campus. Insulin levels were measured by enzyme linked immunosorbent assay (ELISA) technique using insulin specific kits (Labor Diagnostika Nord, Nordhorn, Germany) with sensitivity of 1.76 μIU/ml.

#### Homoeostatic model assessment - insulin resistance (HOMA-IR)

Insulin resistance was assessed using HOMA-IR, one of the most widely used indices based on fasting parameters [40]. It is considered to be a reliable surrogate measure of in vivo insulin sensitivity in humans [41] as well as a practical and convenient tool [42]. The HOMA-IR formula used was the following:

HOMA-IR = {[fasting insulin (μU/mL)] × [fasting glucose (mmol/L)]}/22.5 [40]. A person with HOMA-IR levels ≥2.5 was considered to be insulin resistant [43].

### Statistical analyses

Quantitative and qualitative measurements were summarized as mean ± standard deviation and n (%), respectively. Comparisons of continuous and categorical variables were performed using independent sample T Test (across 2 groups) - One Way Analysis of Variance (ANOVA)/ Kruskal Wallis test (across > 2 groups) test and the chi square test, respectively. Two logistic regression models, stratified by gender (because of interaction between gender and body mass index (BMI), gender and TG), were performed, where HOMA-IR categories (categorical) were used as the dependent variable and dairy intake (continuous) was used as the main independent variable, controlling for important confounders having a *p*-value < 0.20 among males and a *p*-value < 0.05 among females in the bivariate analysis, in addition to age. Among males, these confounders included education, income, BMI, PBF, risky waist circumference, physical activity (PA) and TG levels. However, education and income were removed from the model because of high correlation (*ρ* > 0.80). Among females, confounders that were controlled for were marital status, BMI, PBF, risky waist circumference, TG, HDL and CRP levels. Statistical analyses were performed using the Statistical Package for Social Sciences (SPSS) version 23 for Windows. A *p*-value of less than 0.05 was considered statistically significant.

## Results

**Fig. 1:**
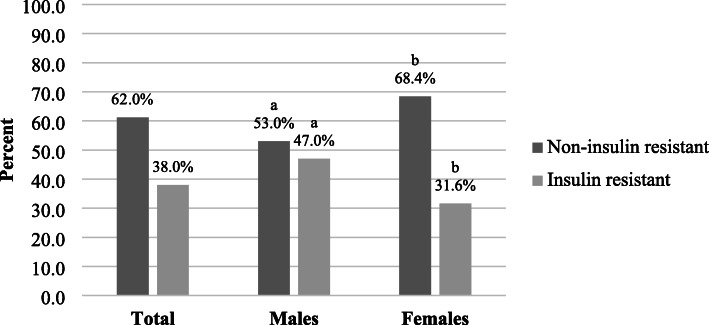
Insulin resistance^1^ in the sample and across genders. ^1^ Defined as HOMA-IR ≥ 2.5 [43]

A total of 286 subjects (46.9% men and 53.1% women) with a mean age of 41.2 ± 11.0 years participated in the study. Characteristics of study population stratified by gender were summarized in Table 1. The majority of participants were healthy, not suffering from medical morbidity (65.4%), non-smokers (63.6%), and did not drink alcohol (75.2%). More than half of the participants (64.0%) had a low level of PA. Subjects were primarily married (63.3%), holding a university degree (80.1%), with an income of less than $4000 per month (58.0%). More than one third of the participants were of normal weight (39.2%), while the majority was overweight and obese (60.1%) (Table 1). Mean glucose and insulin concentrations were 5.0 ± 0.9 mmol/L and 12.1 ± 8.0 uIU/ mL, respectively. In the total sample, more than one third of participants (38.0%) were insulin resistant (Fig. 1).

**Table 1:** Sample characteristics (socio-demographic, dietary, lifestyle, anthropometric, and biochemical factors)

	Total (*n* = 286)		Men (*n* = 134)		Women (*n* = 152)		
Characteristics	n or mean	% or SD	n or mean	% or SD	n or mean	% or SD	*P*-value
Age (years)	41.2	11.0	43.7	11.8	39.0	9.7	< 0.001
Medical morbidity							
- No	187	65.4	83	61.9	104	68.4	0.250
- Yes	99	34.6	51	38.1	48	31.6	
Smoking							
- No	182	63.6	73	54.5	109	71.7	0.003
- Yes	104	36.4	61	45.5	43	28.3	
Alcohol drinking							
- No	215	75.2	89	66.4	126	82.9	< 0.001
- Yes	71	24.8	45	33.6	26	17.1	
PA^1^ level							
- Low	183	64.0	82	61.2	101	66.4	0.356
- Moderate/High	103	36.0	52	38.8	51	33.6	
Marital status							
- Single/ Separated/ Divorced	105	36.7	45	33.6	60	39.5	0.302
- Married	181	63.3	89	66.4	92	60.5	
Education level							
- High school	57	19.9	37	27.6^a^	20	13.2^a^	0.003
- Bachelor degree	76	26.6	27	20.1^a^	49	32.2^a^	
- Graduate	153	53.5	70	52.2	83	54.6	
Income ($)							
- < 2250	85	29.7	44	32.8	41	27.0	0.109
- 2250–4000	81	28.3	30	22.4	51	33.6	
- > 4000	120	42.0	60	44.8	60	39.5	
BMI^2^							
- Underweight	2	0.7	0	0.0	2	1.3	< 0.001
- Normal	112	39.2	25	18.7^a^	87	57.2^a^	
- Overweight	107	37.4	69	51.5^a^	38	25.0^a^	
- Obese	65	22.7	40	29.9^a^	25	16.4^a^	
PBF^3^	30.6	8.0	27.1	6.7	33.7	7.7	< 0.001
Waist circumference risky^4^							
- No	152	53.1	74	55.2	78	51.3	0.509
- Yes	134	46.9	60	44.8	74	48.7	
Calories (Cal)	1942.2	811.5	2165.9	944.3	1744.9	611.6	< 0.001
Dairy product intake (serving/ day)	2.2	1.0	2.2	1.0	2.2	1.0	0.999
Glucose (mmol/L)	5.0	0.9	5.3	1.2	4.8	0.5	< 0.001
Insulin (uIU/ ml)	12.1	8.0	12.3	6.8	11.8	8.9	0.604
Hypertriglyceridemia							
- Normal TG^5^ levels	204	71.3	75	56.0	129	84.9	< 0.001
- Hypertriglyceridemia	82	28.7	59	44.0	23	15.1	
Cholesterol^6^							
- Desirable	185	64.7	90	67.2	95	62.5	0.410
- High	101	35.3	44	32.8	57	37.5	
HDL^7^							
- Normal	219	76.6	100	74.6	119	78.3	0.466
- Low	67	23.4	34	25.4	33	21.7	
LDL^8^							
- Optimal	98	34.5	47	35.6	51	33.6	0.717
- Above optimal	186	65.5	85	64.4	101	66.4	
CRP^9^							
- Low/ moderate	124	43.4	46	34.3	78	51.3	0.004
- High	162	56.6	88	65.7	74	48.7	

^1^ Physical activity

^2^ Body Mass Index - BMI < 18.5 kg/m^2^, normal weight 18.5–24.9 kg/m^2^, overweight 25–29.9 kg/m^2^, and obese ≥30 kg/m^2^ [44]

^3^ Percent body fat

^4^ Risky waist circumference: > 102 cm in men, > 88 cm in women [44]

^5^ Triglycerides – Normal levels: < 150 mg/ dL [45]

^6^ Desirable levels: < 200 mg/dL [45]

^7^ High Density Lipoprotein – Normal levels: ≥40 mg/ dL, ≥50 mg/ dL [45]

^8^ Low Density Lipoprotein – Optimal levels: < 100 mg/dL [45]

^9^ C-Reactive Protein – Low/ moderate levels: < 3 mg/L [46]

Columns with superscripts without a common symbol differ, *P* < 0.05

**Fig. 2:**
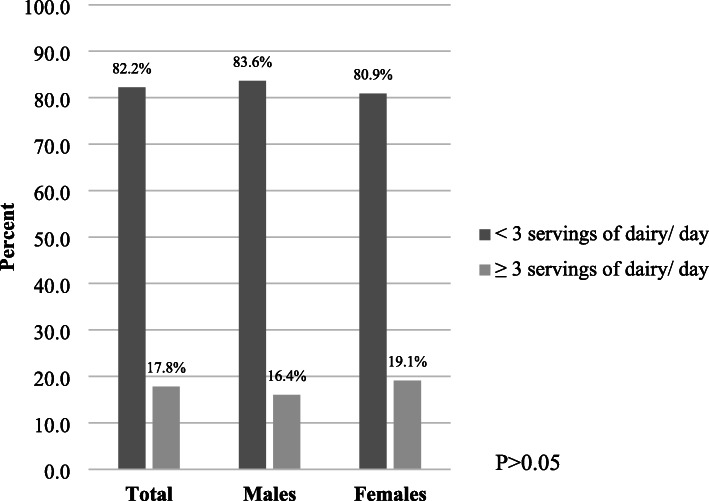
Proportion of individuals meeting the USDA recommendation for dairy intake [47]

The average dairy product intake in the total sample was 2.2 ± 1.0 servings per day. More than 80% of participants were not meeting the recommendation of ≥3 servings of dairy/ day with no statistically significant difference across genders (*p* = 0.557) (Fig. 2).

Across genders, men were significantly older (43.7 ± 11.8 years) than women (age: 39.0 ± 9.7 years) (*p* < 0.001). More men (45.5%) were smokers (*p* = 0.003) and alcohol drinkers (33.6%) compared to women (28.3% were smokers and 17.1% were alcohol drinkers, *p* < 0.001). More than half of women were of a healthy weight (57.2%) whereas more than half of men were overweight (51.5%) and obese (29.9%) (*p* < 0.001). Men had significantly lower PBF (27.1 ± 6.7%) than women (PBF: 33.7 ± 7.7%, *p* < 0.001). Men had higher glucose levels (5.3 ± 1.2 mmol/L) than women 4.8 ± 0.5 mmol/L, *p* < 0.001) (Table 1). A higher proportion of men (47.0%) were insulin resistant compared to women (31.6%, *p* < 0.01) (Fig. 1).

Table 2 summarizes the socio-demographic, dietary, lifestyle, anthropometric, and biochemical factors associated with dairy consumption. A higher proportion of married participants (75.3%) were in quartile 3 of dairy intake compared to quartile 4 (50.7%, *p* = 0.024) while a higher proportion of single participants were in quartile 4 (49.3%) of dairy intake. Individuals in quartile 3 of dairy product intake had lower BMI (25.6 ± 4.3 kg/m^2^), PBF (28.4 ± 7.1%) and waist circumference (92.8 ± 11.6 cm) than individuals in quartile 2 (BMI: 28.0 ± 5.2 kg/m^2^, *p* = 0.021; PBF: 31.8 ± 8.1%, *p* = 0.039; waist circumference: 98.8 ± 12.0 cm, *p* = 0.019). Subjects in the lowest quartile of dairy intake had lower intake of calcium (619.5 ± 435.2 mg), magnesium (248.5 ± 142.0 mg) and potassium (2294.5 ± 1049.1 mg) compared to individuals with the highest intake of dairy (calcium: 887.4 ± 525.3 mg, *p* = 0.011; magnesium: 319.7 ± 154.4 mg, *p* = 0.025; potassium: 2890.8 ± 1288.1 mg, *p* = 0.026). Normal TG levels were more common among individuals in quartile 3 of dairy intake (78.1%) compared to individuals in quartile 2 (57.1%) (p = 0.025) (Table 2).

**Table 2:** Socio-demographic, dietary, lifestyle, anthropometric, and biochemical factors associated with dairy intake

	Dairy intake quartiles								
Characteristic	Quartile 1 (≤1.55 serv/ d) *n* = 72		Quartile 2 (1.56–2.15 serv/d) *n* = 70		Quartile 3 (2.16–2.73 serv/d) *n* = 73		Quartile 4 (≥2.74 serv/ d) *n* = 71		
	n or mean	% or SD	n or mean	% or SD	n or mean	% or SD	n or mean	% or SD	*P*-value
Age (years)	42.3	12.3	42.6	12.1	39.9	9.6	40.1	9.5	0.443
Gender									
- Male	32	44.4	36	51.4	36	49.3	30	42.3	0.674
- Female	40	55.6	34	48.6	37	50.7	41	57.7	
Marital status									
- Single/ Separated/Divorced	26	36.1	26	37.1	18	24.7^a^	35	49.3^a^	0.024
- Married	46	63.9	44	62.9	55	75.3^a^	36	50.7^a^	
Education level									
- High school	17	23.6	14	20.0	12	16.4	14	19.7	0.446
- Bachelor degree	13	18.1	24	34.3	21	28.8	18	25.4	
- Graduate	42	58.3	32	45.7	40	54.8	39	54.9	
Income ($)									
- < 2250	17	23.6	23	32.9	18	24.7	27	38.0	0.293
- 2250–4000	19	26.4	19	27.1	21	28.8	22	31.0	
- > 4000	36	50.0	28	40.0	34	46.6	22	31.0	
Medical morbidity									
- No	42	58.3	41	58.6	49	67.1	55	77.5	0.053
- Yes	30	41.7	29	41.4	24	32.9	16	22.5	
PA^1^ level									
- Low	46	63.9	47	67.1	47	64.4	43	60.6	0.880
- Moderate/ High	26	36.1	23	32.9	26	35.6	28	39.4	
BMI^2^ (Kg/m^2^)	26.1	4.3	28.0^a^	5.2	25.6^a^	4.3	27.0	5.2	0.021
PBF^3^	31.5	8.2	31.8^a^	8.1	28.4^a^	7.1	30.8	8.1	0.039
Waist circumference (cm)	94.8	10.6	98.8^a^	12.0	92.8^a^	11.6	94.7	12.5	0.019
Calcium (mg)	619.5^a^	435.2	744.0	415.7	815.4	595.7	887.4^a^	525.3	0.011
Magnesium (mg)	248.5^a^	142.0	264.2	140.5	285.6	148.3	319.7^a^	154.4	0.025
Potassium (mg)	2294.5^a^	1049.1	2474.7	1143.2	2617.3	1308.5	2890.8^a^	1288.1	0.026
Glucose (mmol/L)	5.0	0.5	5.2	1.3	5.0	0.5	5.0	1.1	0.363
Insulin (uIU/ ml)	12.1	6.2	12.9	10.0	11.3	7.1	12.0	8.3	0.729
HOMA-IR^4^									
- Non-insulin resistant	43	59.7	41	58.6	47	64.4	44	62.0	0.897
- Insulin resistant	29	40.3	29	41.4	26	35.6	27	38.0	
Hypertriglyceridemia									
- Normal TG^5^ levels	54	75.0	40	57.1^a^	57	78.1^a^	53	74.6	0.025
- Hypertriglyceridemia	18	25.0	30	42.9^a^	16	21.9^a^	18	25.4	
Cholesterol^6^									
- Desirable	44	61.1	40	57.1	51	69.9	50	70.4	0.259
- High	28	38.9	30	42.9	22	30.1	21	29.6	
HDL^7^									
- Normal	55	76.4	49	70.0	62	84.9	53	74.6	0.197
- Low	17	23.6	21	30.0	11	15.1	18	25.4	
LDL^8^									
- Optimal	25	34.7	19	27.5	29	39.7	25	35.7	0.492
- Above optimal	47	65.3	50	72.5	44	60.3	45	64.3	
CRP^9^									
- Low/ moderate	29	40.3	25	35.7	35	47.9	35	49.3	0.309
- High	43	59.7	45	64.3	38	52.1	36	50.7	

^1^ Physical activity

^2^ Body Mass Index - BMI < 18.5 kg/m^2^, normal weight 18.5–24.9 kg/m^2^, overweight 25–29.9 kg/m^2^, and obese ≥30 kg/m^2^ [44]

^3^ Percent body fat

^4^ Homeostatic Model Assessment for Insulin Resistance - HOMA-IR levels ≥2.5 indicates insulin resistance [43]

^5^ Triglycerides – Normal levels: < 150 mg/ dL [45]

^6^ Desirable levels: < 200 mg/dL [45]

^7^ High Density Lipoprotein – Normal levels: ≥40 mg/ dL, ≥50 mg/ dL [45]

^8^ Low Density Lipoprotein – Optimal levels: < 100 mg/dL [45]

^9^ C-Reactive Protein – Low/ moderate levels: < 3 mg/L [46]

Columns with superscripts without a common symbol differ, *P* < 0.05

Table 3 summarizes the socio-demographic, dietary, lifestyle, anthropometric, and biochemical factors associated with HOMA-IR across genders. A higher proportion of obese males (42.9%) were insulin resistant compared to those who weren’t (18.3%) (*p* < 0.001). In addition, males with HOMA-IR ≥2.5 had significantly higher PBF (29.4 ± 6.2%) compared to those with HOMA-IR < 2.5 (25.0 ± 6.5%, *p* < 0.001). A higher proportion of males having risky waist circumference were insulin resistant (66.7%), compared to those having a healthy waist circumference (25.4%) (*p* < 0.001). Furthermore, males with HOMA-IR ≥2.5 had higher glucose (5.6 ± 1.6) and insulin levels (16.3 ± 8.2) compared to those with HOMA-IR < 2.5 (glucose: 5.0 ± 0.5, *p* = 0.003, insulin: 8.8 ± 1.3, *p* < 0.001).

**Table 3:** Socio-demographic, lifestyle, anthropometric, and biochemical factors associated with HOMA-IR^1^

	HOMA-IR categories									
	Men (*n* = 134)					Women (*n* = 152)				
Characteristic	HOMA-IR < 2.5 *N* = 71		HOMA-IR ≥2.5) *N* = 63			HOMA-IR < 2.5 *N* = 104		HOMA-IR ≥2.5) *N* = 48		
	n or mean	% or SD	n or mean	% or SD	*P*-value	n or mean	% or SD	n or mean	% or SD	*P*-value
Age (years)	43.9	12.8	43.4	10.5	0.807	39.0	9.0	39.0	11.1	0.978
Medical morbidity										
- No	46	64.8	37	58.7	0.471	75	72.1	29	60.4	0.149
- Yes	25	35.2	26	41.3		29	27.9	19	39.6	
Smoking										
- No	38	53.5	35	55.6	0.813	76	73.1	33	68.8	0.582
- Yes	33	46.5	28	44.4		28	26.9	15	31.3	
Alcohol drinking										
- No	47	66.2	42	66.7	0.954	83	79.8	43	89.6	0.137
- Yes	24	33.8	21	33.3		21	20.2	5	10.4	
PA^2^ level										
- Low	39	54.9	43	68.3	0.114	67	64.4	34	70.8	0.437
- Moderate/High	32	45.1	20	31.7		37	35.6	14	29.2	
Marital status										
- Single/ Separated/ Divorced	24	33.8	21	33.3	0.954	32	30.8^a^	28	58.3^a^	0.001
- Married	47	66.2	42	66.7		72	69.2^a^	20	41.7^a^	
Education level										
- High school	19	26.8	18	28.6	0.124	10	9.6	10	20.8	0.154
- Bachelor degree	10	14.1	17	27.0		34	32.7	15	31.3	
- Graduate	42	59.2	28	44.4		60	57.7	23	47.9	
Income ($)										
- < 2250	20	28.2	24	38.1	0.095	25	24.6	16	33.3	0.467
- 2250–4000	13	18.3	17	27.0		37	35.6	14	29.2	
- > 4000	38	53.5	22	34.9		42	40.4	18	37.5	
BMI^3^ (Kg/m^2^)										
- Underweight						2	1.9	0	0.0	< 0.001
- Normal	21	29.6^a^	4	6.3^a^	< 0.001	69	66.3^a^	18	37.5^a^	
- Overweight	37	52.1	32	50.8		25	24.0	13	27.1	
- Obese	13	18.3^a^	27	42.9^a^		8	7.7^a^	17	35.4^a^	
PBF^4^	25.0	6.5	29.4	6.2	< 0.001	31.4	6.6	38.7	7.5	< 0.001
Waist circumference risky^5^										
- No	53	74.6^a^	21	33.3^a^	< 0.001	67	64.4^a^	11	22.9^a^	< 0.001
- Yes	18	25.4^a^	42	66.7^a^		37	35.6^a^	37	77.1^a^	
Sweets or carbonated beverages										
- < 1 beverage/ day	48	67.6	38	60.3	0.380	80	76.9	35	72.9	0.593
- ≥1 beverage/ day	23	32.4	25	39.7		24	23.1	13	27.1	
Dairy product intake	2.1	1.1	2.3	0.9	0.344	2.2	0.9	2.1	1.1	0.534
Glucose (mmol/L)	5.0	0.5	5.6	1.6	0.003	4.7	0.4	5.1	0.6	< 0.001
Insulin (uIU/ ml)	8.8	1.3	16.3	8.2	< 0.001	8.5	1.6	19.0	13.1	< 0.001
Hypertriglyceridemia										
- Normal TG^6^ levels	44	62.0	31	49.2	0.137	99	95.2^a^	30	62.5^a^	< 0.001
- Hypertriglyceridemia	27	38.0	32	50.8		5	4.8^a^	18	37.5^a^	
Cholesterol^7^										
- Desirable	51	71.8	39	61.9	0.222	69	66.3	26	54.2	0.149
- High	20	28.2	24	38.1		35	33.7	22	45.8	
HDL^8^										
- Normal	55	77.5	45	71.4	0.423	87	83.7^a^	32	66.7^a^	0.018
- Low	16	22.5	18	28.6		17	16.3^a^	16	33.3^a^	
LDL^9^										
- Optimal	28	40.0	19	30.6	0.263	39	37.5	12	25.0	0.129
- Above optimal	42	60.0	43	69.4		65	62.5	36	75.0	
CRP^10^										
- Low/ moderate	26	36.6	20	31.7	0.553	61	58.7^a^	17	35.4^a^	0.008
- High	45	63.4	43	68.3		43	41.3^a^	31	64.6^a^	

^1^ Homeostatic Model Assessment for Insulin Resistance - HOMA-IR levels ≥2.5 indicates insulin resistance [43]

^2^ Physical activity

^3^ Body Mass Index - BMI < 18.5 kg/m^2^, normal weight 18.5–24.9 kg/m^2^, overweight 25–29.9 kg/m^2^, and obese ≥30 kg/m^2^ [44]

^4^ Percent body fat

^5^ Risky waist circumference: > 102 cm in men, > 88 cm in women [44]

^6^ Triglycerides – Normal levels: < 150 mg/ dL [45]

^7^ Desirable levels: < 200 mg/dL [45]

^8^ High Density Lipoprotein – Normal levels: ≥40 mg/ dL, ≥50 mg/ dL [45]

^9^ Low Density Lipoprotein – Optimal levels: < 100 mg/dL [45]

^10^ C-Reactive Protein – Low/ moderate levels: < 3 mg/L [46]

Columns with superscripts without a common symbol differ, *P* < 0.05

Among females, a higher proportion of married females (69.2%) were insulin resistant compared to those who weren’t (41.7%, *p* < 0.001). Moreover, a higher proportion of obese females were insulin resistant (35.4%) than non-insulin resistant (7.7%, p < 0.001). In addition, females with HOMA-IR ≥2.5 had significantly higher PBF (38.7 ± 7.5%) compared to those with HOMA-IR < 2.5 (31.4 ± 6.6%, *p* < 0.001). A higher proportion of females having risky waist circumference were insulin resistant (77.1%), compared to those having a healthy waist circumference (35.6%, *p* < 0.001). Furthermore, females with HOMA-IR ≥2.5 had higher glucose (5.1 ± 0.6) and insulin levels (19.0 ± 13.1) compared to those with HOMA-IR < 2.5 (glucose: 4.7 ± 0.4, *p* < 0.001, insulin: 8.5 ± 1.6, p < 0.001). Normal TG (95.2%) and HDL levels (83.7%) and low/moderate CRP levels (58.7%) were more common among individuals who weren’t insulin resistant compared to those who were (normal TG: 62.5%, *p* < 0.001, normal HDL: 66.7%, *p* = 0.018 and low/moderate CRP: 35.4%, *p* = 0.008) (Table 3).

Tables 4 and 5 examined the association between dairy intake and HOMA-IR, after controlling for confounding variables among males and females, respectively. Among males, the odds of having insulin resistance were 3.9 times higher (95%CI 1.4–11.0; *p* = 0.009) for those having a risky waist circumference compared to those having a healthy waist circumference. Among females, being married had lower odds of having insulin resistance than single females (OR: 0.2, 95%CI 0.1–0.5, *p* = 0.002). As PBF increased by 1 unit, the odds of having insulin resistance increased by 1.2 times (95%CI 1.0–1.3, *p* = 0.008). Finally, the likelihood of having insulin resistance were 8.7 times higher among those suffering from hypertriglyceridemia compared to those with normal TG levels (95%CI: 2.1–35.9,*p* = 0.003). Dairy product intake was not associated with HOMA-IR neither among males (95%CI 0.6–1.4, *p* = 0.777) nor among females (95%CI 0.6–1.6, *p* = 0.968), after controlling for important confounding variables (Tables 4 and 5).

**Table 4:** Logistic regression for dairy product intake and HOMA-IR^1^ after controlling for confounders among males

	HOMA-IR			
Characteristic	Odds Ratio	95% Confidence interval		*P*-value
		Lower	Upper	
Age (years)	0.971	0.936	1.008	0.123
PA^2^ level	0.581	0.249	1.357	0.210
BMI^3^ (Kg/m^2^)	1.378	0.590	3.220	0.458
PBF^4^	1.039	0.951	1.134	0.396
Waist circumference risky^5^	3.927	1.398	11.034	0.009
Dairy product intake (serving/ day)	0.941	0.616	1.436	0.777
Hypertriglyceridemia^6^	0.953	0.412	2.207	0.911

^1^ Homeostatic Model Assessment for Insulin Resistance - HOMA-IR levels ≥2.5 indicates insulin resistance [43]

^2^ Physical activity

^3^ Body Mass Index - BMI < 18.5 kg/m^2^, normal weight 18.5–24.9 kg/m^2^, overweight 25–29.9 kg/m^2^, and obese ≥30 kg/m^2^ [44]

^4^ Percent body fat

^5^ Risky waist circumference: > 102 cm in men, > 88 cm in women [44]

^6^ Triglycerides – Normal levels: < 150 mg/ dL [45]

**Table 5:** Logistic regression for dairy product intake and HOMA-IR^1^ after controlling for confounders among females

	HOMA-IR			
Characteristic	Odds Ratio	95% Confidence interval		*P*-value
		Lower	Upper	
Age (years)	0.946	0.891	1.005	0.073
Marital status	0.194	0.070	0.535	0.002
BMI^2^ (Kg/m^2^)	1.025	0.435	2.431	0.955
PBF^3^	1.162	1.040	1.298	0.008
Waist circumference risky^4^	1.461	0.3620	5.901	0.594
Dairy product intake (serving/ day)	1.009	0.637	1.600	0.968
Hypertriglyceridemia^5^	8.715	2.117	35.875	0.003
HDL^6^	3.056	0.951	9.823	0.061
CRP^7^	0.756	0.258	2.212	0.609

^1^ Homeostatic Model Assessment for Insulin Resistance - HOMA-IR levels ≥2.5 indicates insulin resistance [43]

^2^ Body Mass Index - BMI < 18.5 kg/m^2^, normal weight 18.5–24.9 kg/m^2^, overweight 25–29.9 kg/m^2^, and obese ≥30 kg/m^2^ [44]

^3^ Percent body fat

^4^ Risky waist circumference: > 102 cm in men, > 88 cm in women [44]

^5^ Triglycerides – Normal levels: < 150 mg/ dL [45]

^6^ High Density Lipoproteins – Normal levels: ≥40 mg/ dL, ≥50 mg/ dL [45]

^7^ C-Reactive Protein – Low/ moderate levels: < 3 mg/L [46]

## Discussion

The present study showed that the prevalence of insulin resistance is high in a sample of Lebanese adults, with a higher prevalence among men as compared to women. Dairy product intake was not significantly associated with insulin resistance.

In our study, the prevalence of insulin resistance was 38.0%. Our findings are in line with another local study by Naja et al., 2012 (44.6%), among a national random sample of 308 Lebanese adults [10] with similar characteristics of the study populations including mean age, gender distribution, average BMI, and smoking status. Internationally, the prevalence of insulin resistance among adults varies across different countries, ranging from 15.5% in Denmark [6] to 46.5% in Venezuela [9]. Higher prevalence rate of insulin resistance is reported in Lebanon since the Arab ethnicity is associated with higher risk of developing type 2 diabetes mellitus when compared with other ethnicities [48].

In the current study, the prevalence of insulin resistance was higher among men compared to women, while Naja et al., 2012 did not report on gender differences, other studies did report similar trends [6, 49]. The findings of our study are concordant with those of Friedrich et al., 2012, since in both studies, male participants had significantly higher age and BMI than women, which could explain the difference in prevalence rates of insulin resistance.

The average dairy product intake in this study was 2.2 ± 1.0 servings/day which was in line with another study conducted in Lebanon by Nasreddine et al., 2006 that reported a consumption of 2.06 servings per day among a random sample of 444 Lebanese adults aged between 25 and 54 years, recruited in Beirut and its suburbs [25]. In contrast, Farhat et al. 2016 reported a lower consumption of dairy products of 1.4 servings/day among a convenient sample of 615 adults aged between 19 to 70 years recruited from different regions across Lebanon [26]. The average consumption of dairy products differed between studies due to difference in the study populations including different age ranges and areas from which the samples were selected and different dietary assessment tools.

The consumption of dairy products was below the recommendation of 3 cups per day set by the United States Department of Agriculture (USDA) myplate [47] and the Lebanese Food-Based Dietary Guidelines [50]. Several countries in the Middle East reported dairy product consumption below the USDA myplate recommendation [51, 52]. For instance, the average intake of dairy products was 2.2 ± 1.1 servings/ day among a sample of 126 Emirati adults with an average age of 37.0 ± 11 years [51]. Further, lower intakes of dairy products (0.7–0.85 servings/day) have been reported among a sample of 486 Iranian women aged between 40 and 60 years [52].

Several anthropometric measures including BMI, PBF and waist circumference were inversely associated with dairy consumption. Our results were concordant with the literature [53, 54]. For instance, Mimiran et al., 2005 reported a significant inverse correlation between the number of servings of dairy per day and BMI among a sample of 462 healthy Iranians, aged > 16 years, after controlling for important confounders (*r* = − 0.38, *p* < 0.05) [53]. Moreover, Shin et al., 2017, reported that those who consumed ≥2 servings of milk per day had lower BMI than those who consumed none or rarely milk (*p* < 0.0001) among 86,738 Korean women aged between 40 and 69 years [54]. Further, Shin et al., 2017, reported that higher milk consumption was associated with reduced odds of hypertriglyceridemia, which was also observed by our study and by others in the literature [54–56]. The mechanism by which dairy consumption affects body weight, fat percentage and triglycerides is not fully elucidated, however few mechanisms were suggested [57, 58]. Dairy products are an important source of calcium and individuals consuming dairy products tend to have higher calcium intakes, as evident by our study and by others [59, 60]. Several studies found an inverse relationship between body weight or body fat and calcium [61–63]. It could be that calcium affects body weight and fat mass through inhibiting fat absorption which has been shown to cause a reduction in TG level [64]. Further, calcium helps in the regulation of adipocyte metabolism, decreasing fatty acid synthesis, increasing lipolysis, and thus depleting TG stores [65].

In our study, a higher proportion of married females were not insulin resistant compared to those who were single. The association between marital status and insulin resistance was not thoroughly investigated in the literature except in one study that showed opposite results [9]. Bermudez et al., 2016 showed that married individuals had higher HOMA-IR values compared to single individuals among 2026 participants with an average age of 39.7 ± 15.3 years [9]. In this study, a higher proportion of married participants were males, inactive and had elevated BMI which would explain their results [9]. While in the present study, no statistical difference was observed between single and married females on these same parameters, however married females were more likely to have normal HDL levels (*p* = 0.043), which could explain the association observed in our study.

In our study, participants with higher adiposity were more likely to be insulin resistant. Similar results were observed previously in the literature [13, 14, 66]. In case of high PBF, the liver is directly exposed to free fatty acids and pro-inflammatory cytokines released from visceral fat tissue into the portal vein of obese subjects which will lead to the development of hepatic insulin resistance and type 2 diabetes [15].

Our study found that females with normal TG levels were less likely to be insulin resistant. Likewise, higher risk of hypertriglyceridemia were observed among 990 Thai women aged ≥35 years in the highest quartile of HOMA-IR values compared to those in the lowest quartile [7]. Further, Keska et al., 2013 reported that individuals with higher HOMA-IR had higher TG levels compared to their counterparts with lower HOMA-IR (*p* < 0.01) among 87 young men with an average age of 19.8 ± 0.8 years [67].

Our study did not find an association between dairy product intake and insulin resistance, before and after controlling for confounding variables. The association between dairy product intake and insulin resistance is inconsistent in the literature. For instance, Ma et al., 2006 and Akter et al., 2013 reported similar results in 1087 US adults with an average age of 54.8 ± 8.4 years and 496 Japanese adults aged between 20 and 68 years respectively [28, 31], it is important to point out the average or median intake of dairy products reported in all these studies were low. While others [29, 30, 32] that found an improvement in insulin resistance upon dairy product consumption, showed higher intakes of dairy products. In contrast, Tucker et al., 2015 showed an increase in insulin resistance among 272 middle aged non-diabetic American women [33]. This disparity in the results obtained could be due to multiple reasons. For instance, some studies were conducted only on one gender [30, 33] and on people with a specific BMI range [30, 32, 34] or among people reporting specific conditions (i.e. metabolic syndrome) [32, 34]. In contrast, our sample was largely heterogeneous in terms of gender, BMI and metabolic status. Moreover, results of the bivariate analyses showed that dairy consumption was associated with lower adiposity, which is associated with insulin resistance. However, it could be that the study lacked enough power to detect the protective effect of dairy intake against insulin resistance.

Our study has some limitations that warrant mention. First of all, this study had a cross-sectional design and an association derived from a cross-sectional study does not necessarily indicate causality. Second, the sample size was smaller than the calculated one due to exclusion criteria and missing data. In addition, although our sample is representative of NDU employees, yet it is not representative of the general Lebanese population. Moreover, only 20% of participants complied with the USDA recommendation for dairy consumption and thus, the described associations are among participants with low to very-low dairy consumption. For the assessment of energy and nutrient intake, 24-h Multiple-Pass Method recall was used. Although this technique has many memory cues that increase the ability of participant to recall food and beverages consumed in the last 24 h, compared to the usual 24-h recall [39], multiple 24 h recalls could have generated better approximates of usual energy and nutrient intake. On the other hand, the present study has many strengths. To our knowledge, this is the first study in Lebanon and the Middle East to assess the relationship between dairy product intake and insulin resistance. Second, we used two different dietary assessment tools, the FFQ and the 24-h recall Multiple-Pass Method recall. Moreover, this study controlled for many important confounding variables that were not controlled for in previous studies such as TG, HDL, and CRP levels.

## Conclusion

More research focusing on the relationship between dairy intake and insulin resistance is needed, especially in the Arab and Middle-Eastern region. Further, Middle Eastern dairy products are unique, have different nutrient content and thus might have different effect on insulin resistance. Moreover, several nutrients found in dairy products may have an effect on insulin resistance, whether beneficial or harmful. More studies are needed to elucidate which nutrient has the strongest effect. Finally, future studies should also examine the association between dairy products of different fat content and insulin resistance, as this association might be confounded with the fat content of dairy products.

## Supplementary information

**Additional file 1.** Background questionnaire (medical history, socio-demographic and lifestyle questions)

## Data Availability

The datasets used and/or analysed during the current study are available from the corresponding author on reasonable request.

## Abbreviations

ANOVA: One way analysis of variance
BIA: Bioelectrical impedance analysis
BMI: Body mass index
CRP: C - reactive protein
ELISA: Enzyme linked immunosorbent assay
FFQ: Food frequency questionnaire
HDL: High density lipoproteins
HOMA-IR: Homeostatic Model Assessment-Insulin Resistance
LDL: Low density lipoproteins
NDU: Notre Dame University
PA: Physical activity
PBF: Percent body fat
SPSS: Statistical Package for Social Sciences
TG: Triglycerides
US: United States
USDA: United States Department of Agriculture
WHO: World Health Organization
