# Supplementary material for: Evaluation of risk factors for insulin resistance: a cross sectional study among employees at a private university in Lebanon
Source: BMC Endocr Disord. 2020 Jun 10;20:85. doi: 10.1186/s12902-020-00558-9 (PMC7288486; doi:10.1186/s12902-020-00558-9)
Supplement: Supplementary file 1 — Additional file 1. Background questionnaire (medical history, socio-demographic and lifestyle questions) [file 12902_2020_558_MOESM1_ESM.docx]

| Subject Code: --------------------- | | Interviewer Name: -------------------- | Faculty: ----------------- |
| --- | --- | --- | --- |
| Date of birth: ----/ -----/ -------- | | Date of Interview: -----/ -----/ ------- | Time Required: ------- |
| (day/month/ year) | (day/month/year) | |  |

**Background Questionnaire** *(28 Q, 3 pages)*

**Please check one box for each question where there are check boxes. If you do not wish to answer a question, please draw a line through it.**

**Medical history- I**

1. **Have you been recently diagnosed by a doctor with any of the following chronic medical conditions?**

| - No | - Yes (Check all applicable) |
| --- | --- |

| - Heart attack; Heart failure | - Cancer |
| --- | --- |
| - Stroke | - Neurological disease (multiple sclerosis…) |
| - Hypertension | - Kidney disease |
| - Diabetes | - Liver cirrhosis |
| - Asthma | - Thyroid gland disorders |
| - Vitamin D deficiency | - Other: Specify: --------------------------------- |

1. **If your answer is yes to question #2, have you been taking any medication &/or supplement?**

- No
- Yes, Specify name of medication: _________________________________________

1. **Are you pregnant or breastfeeding?**

- No
- Yes

1. **Are you currently taking any oral contraceptive pills?**

- No
- Yes, Specify name: _____________________________________________________

1. **Have you previously taken oral contraceptive pills?**

- No
- Yes, Specify when: _____________________________________________________

1. **Do you have any physical disability?**

- No
- Yes, Specify: _________________________________________________________

**Socio-demographic, plus anthropometric measurements**

1. **Gender**:

| - Male | - Female |
| --- | --- |

1. **Date of Birth:**  ----------/ ---------/ ------------- (day/ month/ year)
2. **Body weight (kg)/Height (cm) (measured by researcher)** *(leave it empty)*

Body weight (kg) ________

Height (cm) _________

1. **Blood pressure measurement (mmHg):** *(leave it empty)* ________
2. **Waist circumference (cm):** *(leave it empty)* ________
3. **Body composition (total body fat %):** *(leave it empty)* ________
4. **Describe your permanent place of residence:**

| - Urban | - Rural |
| --- | --- |

1. **Marital status:**

| - Single | - Separated |
| --- | --- |
| - Married | - Divorced |

1. **Do you have children?**

- No
- Yes, How many? ___________________________________________________

1. **Indicate your level of education**

| - High School (or equivalent) | - University graduate (Master’s, Doctorate degree, or equivalent) |
| --- | --- |
| - University bachelor’s degree (BA, BS) |  |

**Lifestyle questions**

1. **How many meals do you have per day?**

| - One | - Three |
| --- | --- |
| - Two | - Four or more |

1. **How often do you have your meals?**

| - Often | - Occasionally | - Rarely |
| --- | --- | --- |

1. **How often do you have a breakfast?**

| - Daily | - Occasionally | - Rarely |
| --- | --- | --- |

1. **During the past 3 months, have you been taking any vitamin D supplement?**

- No
- Yes If yes, which supplement? *(Include dosage)*____________________________

1. **If your answer is yes to question #20, then how often did you take the vitamin D supplement?**

| - Daily | - Less than 1x/ week |
| --- | --- |
| - Every other day |  |

1. **During the past 3 months, have you been taking any other vitamin or mineral supplement(s)?**

- No
- Yes If yes, which supplement? *(Include dosage)*____________________________

1. **If your answer is yes to question #22, then how often did you take the supplement(s)?**

| - Daily | - Less than 1x/ week |
| --- | --- |
| - Every other day |  |

1. **Have you been recently following a special diet?**

- No
- Yes, Specify: _________________________________________________________

1. **In the past 3 months, on average, how much time per day was you exposed to direct sunlight (between 10:00 am- 4:00 pm)? *(Think about averaging weekdays & weekend days)***

| - 5 min or less | - 31 to 60 min |
| --- | --- |
| - 5 to 15 min | - More than 1 hour |
| - 16 to 30 min |  |

1. **How often do you use sunscreen?**

| - Rarely/ Never | - Sometimes | - Often |
| --- | --- | --- |

1. **Do you smoke?**

| - Daily | - Former daily |
| --- | --- |
| - Occasional | - Former occasional |
|  | - Never smoked |

1. **Do you drink alcohol?**

| - Never/ Occasionally | - 1-2 drinks per day |
| --- | --- |
| - 1-2 drinks per week | - More than 2 drinks per day |
